# Severe adenovirus infection outbreak in Colombia: Experience from a tertiary pediatric hospital in 2022

**DOI:** 10.7705/biomedica.7047

**Published:** 2024-03-31

**Authors:** Natalia Vélez-Tirado, Lina Castaño-Jaramillo, Sonia Restrepo-Gualteros, Catalina Alcalá-Lozano, Erika Ruge, Carolina Puente, Diana Li-Zeng, Diego Chaparro-Arce, María Camila Beltrán-Dimas, Juan Francisco López, Miguel Luengas-Monroy, Diego Galvis-Trujillo, Iván Gutiérrez-Tobar, Pablo Vásquez-Hoyos, Germán Camacho-Moreno

**Affiliations:** 1 Departamento de Inmunología Pediátrica, Fundación Hospital Pediátrico La Misericordia, Bogotá, D.C., Colombia Departamento de Inmunología Pediátrica Fundación Hospital Pediátrico La Misericordia Bogotá, D.C. Colombia; 2 Departamento de Neumología Pediátrica, Fundación Hospital Pediátrico La Misericordia, Bogotá, D.C., Colombia Departamento de Neumología Pediátrica Fundación Hospital Pediátrico La Misericordia Bogotá, D.C. Colombia; 3 Departamento de Pediatría, Universidad Nacional de Colombia, Bogotá, D.C., Colombia Universidad Nacional de Colombia Departamento de Pediatría Universidad Nacional de Colombia Bogotá, D.C. Colombia; 4 Departamento de Cuidado Intensivo Pediátrico, Fundación Hospital Pediátrico La Misericordia, Bogotá, D.C., Colombia Departamento de Cuidado Intensivo Pediátrico Fundación Hospital Pediátrico La Misericordia Bogotá, D.C. Colombia; 5 Departamento de Pediatría, Universidad El Bosque, Bogotá, D.C., Colombia Universidad El Bosque Departamento de Pediatría Universidad El Bosque Bogotá, D.C. Colombia; 6 Departamento de Infectología Pediátrica, Fundación Hospital Pediátrico La Misericordia, Bogotá, D.C., Colombia Departamento de Infectología Pediátrica Fundación Hospital Pediátrico La Misericordia Bogotá, D.C. Colombia; 7 Departamento de Infectología Pediátrica, Clínica Infantil Colsubsidio, Bogotá, D.C., Colombia Departamento de Infectología Pediátrica Clínica Infantil Colsubsidio Bogotá, D.C. Colombia; 8 Departamento de Infectología Pediátrica, Clínica Infantil Santa María del Lago, Bogotá, D.C., Colombia Departamento de Infectología Pediátrica Clínica Infantil Santa María del Lago Bogotá, D.C. Colombia; 9 Departamento de Pediatría, Fundación Universitaria de Ciencias de la Salud, Bogotá, Colombia Departamento de Pediatría Fundación Universitaria de Ciencias de la Salud Bogotá Colombia

**Keywords:** Adenoviridae infections, pediatrics, respiratory insufficiency, shock, intensive care unit, child, infecciones por Adenoviridae, pediatría, insuficiencia respiratoria, choque, unidades de cuidados intensivos, niño

## Abstract

**Introduction.:**

During the SARS-CoV-2 pandemic, many countries experienced decreased respiratory virus circulation, followed by an out-of-season outbreak. In a pediatric hospital in Colombia, we observed a surge in severe adenovirus infections, leading to concerns about the impact of eased public health restrictions and immune debt in children under five years old.

**Objective.:**

To describe the clinical characteristics of patients with severe adenovirus infection in a pediatric hospital in Colombia.

**Materials and methods.:**

We reviewed the data of 227 patients with severe adenovirus infection at the Fundación Hospital Pediátrico La Misericordia.

**Results.:**

A total of 196 patients were included in this study. The median age was two years, and 62% were male. Adenoviruses were isolated from all patients’ samples. Ninetyseven percent were admitted to the pediatric intensive care unit, 94% required respiratory support, and the in-hospital lethality rate was 11%.

**Conclusion.:**

In 2022, there was an outbreak of severe adenovirus infections, affecting mainly children under five years of age, with higher-than-usual mortality.

## Resumen

**Introducción.:**

Durante la pandemia por SARS-CoV-2, muchos países evidenciaron una disminución en la circulación de virus respiratorios, seguida por un brote fuera de la temporada esperada. En un hospital de Colombia, se observó un aumento en los casos de infección grave por adenovirus, lo cual generó preocupación sobre el impacto que tuvo la disminución de los cuidados establecidos durante pandemia y la posible deuda inmunológica en niños menores de cinco años.

**Objetivo.:**

Describir las características clínicas de los pacientes con infección grave por adenovirus en un hospital pediátrico de Colombia.

**Materiales y métodos.:**

Se revisaron 227 pacientes con infección grave por adenovirus en la Fundación Hospital Pediátrico La Misericordia, desde el 1° de enero hasta el 31 de diciembre de 2022.

**Resultados.:**

El estudio incluyó 196 casos. La edad media de los pacientes fue de dos años y el 62 % eran de sexo masculino. Los adenovirus se aislaron a partir de las muestras de todos los pacientes. El 97 % de los pacientes ingres**ó** a la unidad de cuidados intensivos, el 94 % requirió soporte ventilatorio y la tasa de mortalidad fue del 11 %.

**Conclusiones.:**

En el 2022 hubo un brote de adenovirus que afectó principalmente a los niños menores de cinco años, con una mortalidad mayor a lo reportado con anterioridad en Colombia.

Adenovirus is one of the pathological agents causing acute respiratory infections. Most cases occur in children under five years of age and are usually self-limited or mild. However, more aggressive outbreaks have been described, generally associated with serotypes 3 and 7 ^1^. In Colombia and worldwide, the risk of common respiratory infections decreased temporarily due to the non-pharmacological place-preventive strategies during COVID-19.

In 2022, Gutierrez *et al*. reported a significant increase in the circulation of adenovirus in Colombia, making it the second leading cause of severe respiratory infections in pediatric patients. The article described increased admissions to the pediatric intensive care unit (pediatric ICU), the need for extracorporeal membrane oxygenation (ECMO) support, and, unfortunately, deaths. At one pediatric referral center, adenovirus accounted for up to 45.8% of hospitalizations in the pediatric ICU and were responsible for the need for ECMO support in 76% of the patients. This pattern is similar to those documented in other cohorts worldwide ^2^. Some children with sequelae of severe adenovirus respiratory infection may develop persistent wheezing, necrotizing pneumonia, bronchiectasis, atelectasis, and bronchiolitis obliterans, resulting in a disease burden beyond childhood ^2^.

Therefore, we analyzed all severe adenovirus admissions at our center - Fundación Hospital Pediátrico La Misericordia (HOMI) - and established a multicenter Colombian registry (ADENOCOLOMBIA) aiming to characterize the patterns of the severe forms of this adenovirus outbreak. Here, we present some initial results.

## Materials and methods

In this retrospective descriptive study, we reviewed medical records of 227 patients with adenovirus infection, detected by any test (FilmArray^TM^ or antigens) that required staying in the pediatric ICU. Cases with severe adenovirus infection at the HOMI, a pediatric reference hospital in Colombia, included those from January 1^st^, 2022, until December 31^st^, 2022. We included 196 patients in the analysis and excluded 31 because the adenovirus infection was not severe, defining it as requiring ventilatory or vasoactive support. We used central tendency measures to describe the collected data: median and interquartile ranges (IQR).

This retrospective study did not require intervention from the study subjects. We anonymously performed the analyses and did not need informed consent. The study was approved by the HOMI’s Ethics Committee: Record number 69 528-22R.

## Results

The present study included 196 patients infected with adenovirus according to respiratory secretions testing. Infections were detected through a molecular respiratory panel (Film Array™) in 94% of the patients and through antigen detection in 8%. Five patients were tested with both methods. The median age of the patients was two years old, with a male predominance (62%). The median weight was 11 kg (IQR = 7-17) and median height was 85.5 cm (IQR = 66-102). Upper respiratory symptoms were the most common clinical manifestation, observed in 176 patients (89.9%), followed by fever in 137 patients (69.8%). Additionally, diarrhea was reported in 45 patients (23.0%), conjunctivitis in 14 (7.1%), and skin manifestations in 12 (6.1%) patients. Comorbidities were present in 119 patients.

Because some patients underwent double testing, a total of 197 samples were processed: 136 (69%) had another virus in addition to adenovirus, 49 (25%) had two additional viruses, and 12 (6%) had three or more. We performed blood, urine, pleural and peritoneal fluid, and respiratory secretion tests for bacterial detection. We detected a higher percentage (71.5%) of bacteria in respiratory secretions. Chest X-ray revealed lobar or multifocal consolidations in 99 patients (50.5%). However, the most frequent finding was an interstitial pattern in 67.3% of the patients.

In the present study, most patients (97%) were admitted to the pediatric ICU or intermediate care unit, with a median ICU stay of 7 days (IQR = 5-15). A significant number of patients needed respiratory support (94%), with 59% requiring a high-flow nasal cannula, 31%, invasive mechanical ventilation, 7%, high-frequency oscillatory ventilation, and 3%, non-invasive ventilation. Moreover, more than one-third of the patients required vasoactive support (36%), and 15 (8%) were candidates for ECMO therapy.

The median length of hospital stay was 18 days (IQR = 10-30). At hospital discharge, 44% of the patients still required supplemental oxygen support, and 4% had undergone tracheostomy. The in-hospital mortality rate was 11%. Of the 21 deceased patients, 18 had previous comorbidities.

## Discussion

Severe adenovirus respiratory infection outbreaks were described in multiple countries before the SARS-CoV-2 pandemic. However, in 2022, Colombia had an epidemic outbreak like no other that had occurred in over 20 years. The last known adenovirus flare-up occurred in 2003 (unpublished data). We report an unusually large series of severe adenovirus infections in pediatric patients requiring respiratory or vasoactive support, which is atypical at our center.

During the COVID-19 pandemic, non-pharmaceutical interventions were implemented to control the spread of SARS-CoV-2 and reduce burden of other respiratory viruses. However, with the public health restrictions’ lifting in the post-pandemic era, we observed a surge in respiratory virus infections with increased severity ^2^.

This low exposure to viruses resulted in an intense out-of-season outbreak of respiratory syncytial virus (RSV) and influenza, reported already in Asia, Europe, the USA, and some countries of Latin America ^2^. The resurgence of respiratory viruses has affected a more vulnerable children population due to a reduced community level or herd immunity to these pathogens and a reduced transfer of passive immunity from mothers to their infants. This phenomenon is called the “immunity gap” or the “immune debt” ^3^.

In Colombia, the circulation of adenovirus has traditionally been higher during March, October, and December. However, in 2022, adenovirus circulation remained above the expected levels throughout the year, with an increasing positivity rate between 22.7% and 32.5% in the second half of the year. Between 1997 and 2003, adenovirus was found in 2.6% of the analyzed samples; between 2009 and 2011, it was detected in 7.5% of samples from patients with lower respiratory tract disease ^4^. In hospitalized children with community-acquired pneumonia, adenovirus is responsible for more than 10% of the cases, and in 2022, it was the fourth most common respiratory virus in Colombia, with an isolation rate of 16.9% ^1^.

In 2007, Herrera-Rodriguez *et al*. described the epidemiological and clinical characteristics of adenovirus infection in children under five years old in Colombia between 1997 and 2003. During this period, they analyzed 1,743 respiratory samples as part of the influenza epidemiological surveillance program and identified 47 patients positive for adenovirus (2.7%). Of these patients, 71% were male, and 68% were under one year old ^5^. Clinically, ten patients (21%) required ICU admission, with a median stay of 10 days; 4 (8%) required invasive ventilatory support, and one patient (2%) died. Another retrospective study, conducted from 2009 to 2011, reported an ICU or intermediate care admission rate of 28% and a mortality rate of 2.8%. In patients with mixed adenovirus and RSV infection, 57% were admitted to the ICU or intermediate care unit, and the mortality rate was 3.3% ^4^. In our series, we also observed a male predominance, a shorter median stay in the ICU, and higher mortality. Shi *et al*. recently published a series of 168 patients with severe adenovirus infection in a pediatric hospital in China, where 19 subjects (11.3%) required ECMO therapy ^2^. In our case series of 196 patients older than one year, 15 were considered for ECMO therapy (7.7%).

Despite the relevance of adenovirus as an etiological agent, little is known about the serotypes circulating in Colombia, probably because of the limited availability of serotyping methods. Due to the scarcity of epidemiological information about these viruses, it is currently impossible to establish relationships between serotypes and infection severity, meteorological conditions, or virus biological diversity in different country regions. It is essential to assess the situation of adenovirus infection in Colombia to determine the extent of the 2022 outbreak throughout the country. Future studies should include viral genotyping to identify potential adenovirus serotypes responsible for severe disease and to pinpoint whether the circulating strains changed after the COVID-19 pandemic. It is of the utmost importance to analyze biomarkers in these patients to correlate whether certain biochemical phenotypes can predict a more severe disease with higher mortality and worse long-term respiratory consequences.

The single-center character of this study is its main limitation. Additionally, we are a pediatric reference hospital, and our patients frequently have preexisting comorbidities that may have contributed to increased severe adenovirus infection and mortality rate. Another limitation is the missing mortality date data of ten patients referred for ECMO therapy. Furthermore, the molecular respiratory panel test (Film Array™) was not performed for all patients with respiratory symptoms, but it was for those with severe or critical disease. This procedure may have overestimated the severity of adenovirus infection in our series. Adenovirus serotyping was unavailable, thus, we could not determine whether this outbreak was due to serotypes AdV3 and AdV7, as previously described in other series.

To accurately describe the epidemiology of severe adenovirus infection after the COVID-19 pandemic in Colombia, a multicenter study covering different regions of the country is necessary to identify demographic information, predictors of severe disease and mortality, and adenovirus serotypes.
